# The Cure of Pulmonary Tuberculosis upon the Principles of Nutrition*Read at the third annual meeting of the Tri-State Medical Association at Chattanooga, Tenn., October 27, 1891.

**Published:** 1892-02

**Authors:** Karl Von Ruck

**Affiliations:** Asheville, N. C.


### THE CURE OF PULMONARY TUBERCULOSIS UPON THE PRINCIPLES OF NUTRITION.*

BY KARL VON RUCK, B. S., M. D., ASHEVILLE, N. C.

How hopeful such a title sounds, and how pleasant it is to contemplate that such a result may attend our efforts in the treatment of this dread disease.

Yet how difficult it is, how much painstaking effort it requires, both by physician and patient, how frequently our brightening hopes are ruthlessly dashed to pieces by intercurrent relapses, and how hard it seems to regain the lost ground, I have impressed upon me almost every day of my life, and I frequently wonder why I did not choose some easier specialty for my professional labors. Not only is the battle with this disease a difficult one, but disappointment to one's ambition for success arises frequently, even when the cure seems almost completed or within easy reach, by the patient mistaking improvement for real recovery, abandoning his and our efforts too early, and serious or even fatal relapses then follow the return to the climate and conditions of life and habits under which the disease was acquired.

These latter disappointments are specially hard to bear, after months of painstaking efforts and interest in a case, which we have prospects of adding to the list of successful ones, and even with the great preponderance of advanced-stage cases, which seek climatic and other treatment at our hands, I am well satisfied that were it not for this too early abandonment of properlefforts, the permanent results which I have heretofore witnessed could have been easily doubled and perhaps trebled.

Another source for regret, and an important cause for the low per centage of permanent recoveries, is the fact that comparatively few cases come under systematic treatment in the early or incipient stage, the patients either being unwilling to take a serious view of their ailment, as long as the general health has not seriously suffered, or a positive diagnosis with

*Read at the third annual meeting of the Tri-State Medical Association at Chattanooga, Tenn., October 27, 1891.

uncompromising advice having not been given by the patient's physician. Could it be the rule, rather than the exception, that these early-stage cases were placed under conditions of climate, with such management and treatment as even in more advanced cases are still capable of effecting improvement and occasional permament recoveries, I believe the necessity for specifics would be less apparent, and we would succeed even without them in so reducing the mortality, that the disease would be largely robbed of its terrors.

All this will no doubt be more and more appreciated by the patients and their friends in the future, as is already the case with most of the profession, and apart from our employment of prophylactic measures as the "king remedy" for the "king evil" tuberculosis, by which we may hinder the spread of the disease, on our part an early diagnosis and proper advice is all we are responsible for to the patients who consult us.

This responsibility is, I fear, not yet met by the profession in general as fully as it should be, and the time has certainly arrived when failure in diagnosis of the early stage of phthisis should be considered reprehensible and justly deserving of censure. It is the first essential to a cure, and when made may also be coupled with a hopeful prognosis, for even at home much may be accomplished should the patient be poor or entirely unable to secure the additional aids of climate and expert professional management.

At no time will the necessary expense incidental to recovery be less, whether at home or at a resort; on the contrary it will, on an average, cost less than is frequently expended, when by delays more serious and alarming symptoms supervene, and the subsequent unfavorable course leads to the death of the patient.

Mercenary patients may therefore be told that it costs less money and time to recover from the early stage of pulmonary tuberculosis than it will eventually cost to neglect it, and ultimately die from the disease, in spite of more expensive and nevertheless fruitless efforts.

The signs and symptoms of the early stages of consumption are known to every physician who deserves the name, and while their certain recognition requires some training, a strong suspicion of the presence of the disease is justifiable in the majority of cases, even from the history and from clinical observations alone, without physical or microscopical examination, justifying the less expert or less experienced practitioner to call upon his colleagues for aid and counsel, or in the expression of his apprehensions to the patient or the relatives, who then share the responsibility with him.

Let no physician blunder along because he dislikes to admit his inability to make a sure diagnosis until the disease is so far advanced that the neighbors will make it for him. It is no disgrace to be in doubt where greater experience may find an easy certainty; but it is criminal to jeopardize the prospects for recovery of a fellow-being by pretending a knowledge not possessed; and I believe that candor and truthfulness in such matters will earn the less experienced physician more friends, more confidence and a better standing, both as a physician and as a man, in his community, than the ludicrous assumption of knowing all there is to be known in the numerous brances of medical and surgical practice.

In speaking of early diagnosis, I would call your attention to an easily determined condition of the involved apex, which amounts to a sign of present structural changes, and which, with a little practice, is not difficult of recognition. I am not aware of its particular mention for the purpose of diagnosis by American or English authors. In Germany it is well recognized, and von Ziemssen lays particular stress upon its importance, being present sometimes before, and always in addition to, the other physical evidence. I refer to the relatively lower portion of the affected apex which obtains, whenever the usually first involved upper limit expands less and holds less air than is the case on the still healthy opposite side.

To demonstrate it, we percuss upward above the clavicle to a line around the neck where resonance from lung tissue ceases, and mark this line carefully with a colored pencil. In most cases simple inspection is then enough to recognize the line to stand lower on one side than on the other, either anterior or posterior, or both, and this is ususally so, even in advanced cases, where one upper lobe is, as a rule, further advanced than its fellow. To be accurate, we measure from the marked line on the lateral side of the neck to the end of the acromian process, and when errors are excluded, a difference of even a quarter inch being shown, we will rarely seek in vain for other evidences of structural disease on the short side, there existing no appreciable difference between the two sides in health.

The error in diagnosis, most frequently made, is the mistaking of the tubercular disease, for a simple throat or bronchial affection, especially the latter, so long as no marked difference in the percussion note exists. Those less practiced in physical diagnosis, frequently perform percussion, in a way which cannot possibly show fine shades of difference, to bring out which, requires, besides a well-trained ear, a careful application of the finger, or other pleximeter, a practiced, even stroke from the wrist, and the comparison must be made at the same stage of the respiratory act. A distinct crepitation, or fine rales, noted in auscultation, to occur over a circumscribed area involving, for instance, part of an upper lobe, may indeed be due to bronchitis but this kind of circumscribed bronchitis is, as a rule, symptomatic of tubercular disease. The ordinary bronchitis is never so circumscribed; on the contrary, it is diffused; the auscultatory phenomena are practically the same on both sides, and of greatest intensity in the lower lobes. In the absence of symptomatic catarrhal signs, we have the suppressed vesicular, or slightly harsh vesicular respiratory murmur, a change in rhythm or pitch, also circumscribed and easily outlined with a stethoscope.

Take such a case, even before expectoration has made a microscopical examination possible, and watch the temperature, by hourly observations, or by tying a maximum thermometer into the axilla, quite frequently a slight febrile movement will also be found present, and this, when the patient is entirely unaware of its existence; in addition, a more or less hacking cough, or clearing of the throat is seldom denied, if we interrogate carefully. Such symptoms, continuing for a week, a month, or even longer, what can it all mean? How can we still hesitate to diagnose insipient phthisis when, in addition to all this, the patient shows an hereditary, or acquired predisposition, has a quickened pulse, is anæmic, shows loss of strength, or is even losing flesh ?

The diagnosis made, what shall we do with the case to cure it ?

The cure of pulmonary tuberculosis is not an easy matter, and I want to say right here, that there is no one single thing which we can advise or do to the exclusion of many others which will assure success or even improvement; there is no single specific or drug to swallow or to inject, no particular air to breathe, no given place to reside in, and no special mode of life to adopt, which we can recommend as the *sole* road to recovery. The treatment to pursue is based upon much broader principles, in the course of which many therapeutic agents may become valuable at one time or another, as aids to nature's efforts toward a recovery, and has for its object the eradication not only of the tubercular process, but also of the predisposition which made it possible for the tubercular process to become established.

The predisposition is already a departure from the physiological standard of nutritive processes, tending toward or already constituting a pathological state. The resulting biochemical changes for which, indeed, we may as yet have no chemical reaction or microscopical demonstration, influencing the conversion of nutritive as well as waste products. Their elaboration, solubility and excretion become slightly modified, tending under certain conditions to accumulation and retention of substances foreign to the organism in health, or to solubility and excretion of partially elaborated nutritive principles, in either case affecting changes in the fluids and solids of the body, favorable to the localization and growth of pathogenic bacteria, either previously present, or accidentally introduced, at this time, from without.

We have thus a coarse example in diabetes mellitus, and can see some explanation for the frequency with which tuberculosis occurs in the subjects of this disease.

We can indeed also conceive, that under certain conditions similar changes may cause contrary results, so as to diminish the liability for infection from a particular disease germ, or even confer an immunity, the tissues forming a sterile soil for one kind, and a more favorable one for another.

There is a large field for investigation in the line thus only faintly indicated, and the production of highly poisonous substances in the intestinal canal even in apparent health, the formation of toxal-bumins, of leucomains and cadaveric alkaloids, all point to still hidden influences in the chemistry of the living body which probably play a much more important part in the etiology of disease than we at present surmise.

In tuberculosis, for convenience, we call these biochemical changes "predisposition" or the "pre-tubercular stage," the removal of which, as already stated, being as important, if not more so, than are our efforts to eradicate the bacterial manifestations which in my conception are only a step further in the disease process, and become self-limited, if we succeed in returning the organism to a standard of successful resistance.

These are, and ever will remain, the indication for the successful treatment of pulmonary tuberculosis, be that by specifics or other means at our command.

Before, however, considering the utility of any measure which we desire to employ, it is well for us to enquire how nature deals with the disease in her curative efforts.

So many recoveries are now on record, and so frequently are healed tubercular lesions found in post-mortem examinations, that the possibility of a permanent cure requires no adduction of proof.

We have also learned the various histological changes which occur in, and surrounding, the tubercular tissues when the disease follows a favorable course to its permanent arrestment, being essentially the same as follow the introduction of other foreign bodies into living tissue. The foreign substance, in this instance the tubercle, is either extruded by suppuration or it becomes encapsuled.

Were it not for the difficulty or impossibility on the part of nature to entirely expel the tubercular tissue, or the inability on our part to afford surgical aid, and after its accomplishment apply the principles of drainage and antisepsis, no doubt the entire removal (as long as the disease is limited in extent and circumscribed) would be most desirable, as is the case with all foreign bodies and with tubercular deposits in parts accessible and within reach of such aid.

In the pulmonary affection, while such processes may lead to the desired end, the encapsuling is much more safe and desirable, and must be favored by every means at our command.

Had this principle been fully appreciated by the early experimenters with tuberculin, the production of necrosis of tubercular tissue by comparatively large doses of this agent would not only have appeared as undesirable, but as positively dangerous; for with the necrosis and the attending softening and suppuration, come the dangers of inflammatory extension, septic fever, and possibly, absorption and dissemination of the bacilli, leading, under favorable circumstances, to most formidable pneumonic processes, general miliary tuberculosis and exhaustion of the patient, and apart from tuberculin, this occurs frequently, under the inadequate efforts of nature to expel the tubercular tissue in the ordinary course of the disease.

I have never expected good results from such erroneous application of tuberculin, and have only wondered that the results were not much worse under the system of dosage by which also the nutrition of the patient received one blow after another, and the complications which we should ever strive to avoid were purposely favored. It is, however, true, that when the process is strictly limited to a small portion of lung, and the patient is still vigorous, excavation is not usually attended by such dire results, and a real cure may follow the process, the cavity being small, and, if favorably located, may shrink and become lined with fibrous walls, or may cicatrize and become obliterated entirely.

Frequently the tubercular infiltration is much more extensive than we suspect from our physical examinations, which, no matter how carefully made, depend still upon comparatively coarse means ; and a considerable degree of pathological changes must already have occurred before we get evidence of their existence appreciable by the acutest and most practised ear in examination.

When tubercular deposits or other foreign bodies become encapsuled, this occurs by connective-tissue formation upon the periphery, due to the increased nutritive changes in response to the irritation set up in the surrounding tissues.

In the course of this mode of cure a more or less dense fibrous wall finally surrounds the foreign substance, and by the subsequent shrinking and thickening, the vascularity becomes eventually so much reduced that the encapsuled substance is practically shut out, so to say, from the general circulation, and may remain so for an indefinite time, or until it may have undergone transformations of various kinds.

In pulmonary tuberculosis the completion of such a process requires considerable time, and may be interrupted either by the changes which may occur in the encapsuled tubercular tissue itself, or by disturbances in the nutrient activity upon the periphery. For instance, softening may occur in the partially encapsuled tubercle, leading then to suppuration and excavation ; the circulation may become so inadequate as to favor the occurrence of stasis or thrombosis in the nutrient vessels, an l the protecting connective-tissue capsule is then not completed, may become reabsorbed, or break down.

From recent investigations it seems more than probable that the tubercle itself furnishes a certain irritant during its casation which, acting upon the periphery as a stimulant or irritant, favors the connective-tissue formation, but if in excess or if certain injurious influences act from without, the irritation may increase to the production of inflammation with or without suppuration.

According to the quantity and quality of blood freely reaching the locality, and according to otherwise favorable or unfavorable circumstances, the changes for better or worse are rapid or slow, or stationary. In the latter case the tissues themselves become accustomed to the local irritant, and the encapsuling is thus retarded or arrested.

Other things being equal, the logic is unassailable, that the richer the blood is, in its tissue-building elements, and the freer and the less obstructed the circulation to the locality where increased tissue-formation is desired, the surer, quicker and better will be the result.

It follows, therefore, that the process of a cure depends upon the local and general nutrition, and the means which we may hope to be of benefit in eradicating the tubercular tissue by encapsuling and subsequent pressure, atrophy and absorption must be shown to aid directly or indirectly either the local or general nutrition, or both.

To eradicate the predisposition, we have good reason to depend upon the same means until a specific protective remedy shall have been found.

We know that the leucocytes and the blood-serum are antagonistic to pathogenic micro-organisms, and under favorable conditions have the power to destroy them; we know, too, that the blood of certain species of animals has this power to a greater degree than that of others, and it is also probable that a difference in degree exists in the same animal and in different animals of the same species. We believe, therefore, that the susceptibility to tubercular infection is only relative in the human being and is determined by the state and quality of the tissue cells, the serum of the blood and its cellular elements—in other words, by the local and general nutrition.

Empirically we know, also, that anæmia and other nutritive disturbances favor the occurrence and progress of tuberculosis, and that, in the strong and vigorous with healthy functions, the disease is not likely to occur, or is speedily arrested and recovered from, and that, in the favorable course of this disease, relapses follow from even slight nutritive disturbances.

*The key to the arrestment and cure of pulmonary tuberculosis is therefore to be sought in nutrition, local and general, and every remedy must stand or fall as it is useful or useless, to this end, and its greater or lesser value must be estimated by the same standard.*

As the means and measures by which we influence nutrition favorably are many and various, and different individuals respond more or less perfectly to one or the other, so the resources at our command are of the greatest variety, and much will depend upon judgment and adoption of the means for the individual case and stage of the disease reached.

(*To be continued.*)

